# Cross‐Conjugated Polymer Semiconductors

**DOI:** 10.1002/marc.202500281

**Published:** 2025-07-02

**Authors:** Naixin Zhao, Sung Jae Jeon, Yuning Li

**Affiliations:** ^1^ Department of Chemical Engineering and Waterloo Institute for Nanotechnology (WIN) University of Waterloo 200 University Ave West Waterloo Ontario N2L 3G1 Canada

**Keywords:** conjugation switching, cross‐conjugated polymers, cross‐conjugation, linear conjugation, organic electronics, structure–property relationship

## Abstract

Cross‐conjugated polymer semiconductors represent an unconventional yet promising class of materials with distinct structural and electronic characteristics compared to their linear conjugated counterparts. These systems introduce branched π‐electron delocalization, enabling unique optical, electronic, and redox behaviors not accessible through traditional conjugated frameworks. In this review, we provide a comprehensive overview of representative cross‐conjugated polymer systems, emphasizing the structure–property relationships and their implications for optoelectronic performance. We highlight recent advances in the design and application of these materials in organic field‐effect transistors (OFETs), organic light‐emitting diodes (OLEDs), sensors, electrochromic devices, solar cells, and energy storage. While these polymers typically exhibit lower charge carrier mobilities, several notable examples demonstrate that high mobility can be achieved via structural transformation into linearly conjugated systems through electrical, chemical, or tautomerization mechanisms. Overall, cross‐conjugated polymers offer significant potential in emerging applications that demand multifunctionality, environmental responsiveness, and tunable redox behavior.

## Graphical abstract

This review highlights the structural features, electronic properties, and emerging applications of cross‐conjugated polymer semiconductors. By comparing them with linear analogs, the article reveals how cross‐conjugation enables redox activity, conjugation switching, and multifunctionality. Key design strategies and performance benchmarks are discussed, offering insights into future directions for organic electronics beyond high‐mobility devices.

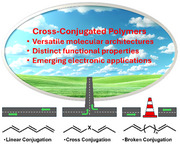

## 1 Introduction

Polymer semiconductors have been extensively studied due to their potential for flexible, lightweight, and solution‐processable electronic applications [1, 2, 3]. Since the discovery of polyacetylene, the pioneering “conductive plastic” in 1987 [4], a wide range of conjugated polymers has been developed. The ability of these materials to transport charge carriers originates from their extended π‐conjugation, where highly delocalized and electron‐dense π bonds facilitate charge transport [5]. As intramolecular charge transport along the polymer backbone is generally much more efficient than intermolecular charge transport via hopping, achieving extended conjugation is essential in the design of high‐mobility conjugated polymers [6].

**FIGURE 1:**
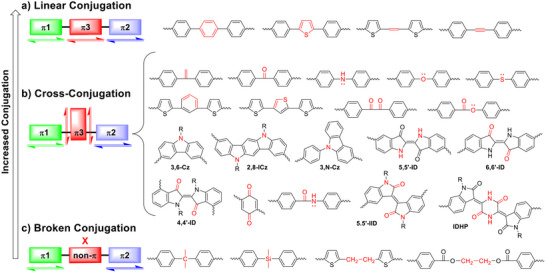
Generalized structures and examples of (a) linear conjugation, (b) cross‐conjugation, and (c) broken conjugation systems, where π1, π2, and π3 represent π‐conjugated units containing 2n π‐electrons. Atoms with lone pairs (e.g., O, S, and N) that participate in π‐conjugation are also considered π‐conjugated units. Abbreviations of core building blocks: **Cz**, carbazole; **ICz**, indolo[3,2‐b]carbazole; **ID**, indigo; **IID**, isoindigo; **IDHP**, 3,6‐bis((*Z*)‐2‐oxoindolin‐3‐ylidene)piperazine‐2,5‐dione. R is hydrogen or an appropriate substituent. Linkages between π‐conjugated units are highlighted in red.

Compounds or building blocks with π‐conjugation can be classified into three categories: linear conjugation, cross‐conjugation, and broken conjugation (Figure 1 and table 1) [7]. In linear conjugation (figure 1a), π‐electrons delocalize continuously over a molecular framework through alternating single and multiple bonds (e.g., double bonds or triple bonds) [8, 9, 10, 11, 12, 13]. Polymers composed of linearly conjugated building blocks form an uninterrupted π‐system, resembling a “highway” that enables efficient intramolecular charge and energy transfer, resulting in enhanced electronic, optical, and conductive properties. As a result, it is widely accepted that monomeric building blocks for conjugated polymers should possess a linear conjugation structure to maximize conjugation length. Over the past decade, numerous building blocks have been developed based on this design principle [1, 14, 15, 16].

**TABLE 1:** Comparison of polymers containing linear conjugation, cross‐conjugation, and broken conjugation.

Feature	Linear conjugation	Cross‐conjugation	Broken conjugation
Definition	Continuous delocalization of π‐electrons along the entire molecular backbone.	Delocalization occurs through multiple pathways, but these pathways do not overlap continuously.	Interrupted conjugation due to non‐conjugated spacers, limiting electron delocalization.
Key Structural Feature	Alternating single and multiple bonds in a linear sequence.	A junction π‐conjugated unit creates branching π‐systems.	Conjugated segments are separated by nonconjugated linkers (e.g., sp^3^‐hybridized carbons, aliphatic chains).
Effect on Optical Properties	Significantly lower HOMO‐LUMO gap due to extended π‐electron delocalization.	Moderately reduced HOMO‐LUMO gap due to weak interactions between branched π‐conjugated systems.	Higher HOMO‐LUMO gap due to the disrupted conjugation length.
Charge Transport	Highly efficient intramolecular charge transport due to continuous conjugation.	Reduced intramolecular charge transport due to conjugation pathway interruptions.	Severely hindered charge transport, with charge movement primarily occurring via intermolecular hopping.

In contrast, cross‐conjugation occurs when π‐electron delocalization branches into two or multiple non‐continuous pathways rather than forming a single extended system [7, 17, 18, 19]. In a cross‐conjugated building block, a central atom or functional group serves as a junction where conjugation diverges into two or more separate paths (FIGURE 1b) [7]. The term “cross” refers to the point where these conjugation pathways meet at a junction atom, which must contain either a π‐electron or a lone pair to maintain conjugation on both sides. Although neighboring conjugation systems exhibit limited interactions in the neutral (pristine) state, cross‐conjugated polymers, and small molecules can transition into a fully, linearly conjugated state under certain conditions—such as in the presence of acids, bases, metal ions, an applied gate voltage, or more generally, in the doped state. However, compared to their linearly conjugated counterparts, they typically exhibit lower charge carrier mobility [18, 20, 21]. Extending the conjugation pathway branched out from the junction point has drawn attention since it may enhance electron transfer [22] and facilitate 2D charge transport [23].

If the atom at the junction of the conjugation systems is replaced with a conjugation‐breaking spacer (e.g., an aliphatic chain), the two conjugation pathways become disconnected, and the polymer has broken conjugation (FIGURE 1c). As a result, the polymer chain consists of localized conjugation segments with little to no optoelectronic interactions. In this case, intramolecular charge transport is significantly restricted, and charge carrier mobility primarily depends on slower intermolecular charge hopping [24, 25, 26]. Recently, notable cross‐hyperconjugation effects have been observed in broken conjugation molecules where the junction is a bis(trimethylsilyl)‐substituted silicon, leading to a significant redshift (or bathochromic shift) in UV–Vis absorption (or a lowered bandgap (*E*_g_)) compared to the one containing the dimethyl‐substituted silicon or dimethyl‐substituted carbon junction [27].

Among the many applications of conjugated polymers, organic field‐effect transistors (OFETs) remain one of the most widely studied. To enable high‐speed electronic applications such as radio‐frequency identification (RFID) tags, conjugated polymers must achieve high charge carrier mobility (e.g., >10 cm^2^ V⁻^1^ s⁻^1^) [10, 12, 28]. Consequently, over the past 30 years, there has been an intense focus on designing high‐mobility conjugated polymers, often referred to as the “mobility hype” [29]. Due to their inherently lower mobility, cross‐conjugated and broken‐conjugated structures have received less research attention compared to linear conjugated building blocks [30].

In recent years, while the development of conjugated polymers with high charge carrier mobility and electrical conductivity remains a central focus, particularly for advancing flexible displays, RFID tags, and thermoelectric devices [2, 31, 32, 33], significant research efforts have also shifted toward a broader set of functional properties tailored to diverse application domains. These include enhanced biodegradable and recyclable conjugated polymers [34], robust mechanical strength and stretchability for wearable and skin‐mounted electronics [35], biocompatibility and aqueous stability for biomedical and implantable devices [36], and high sensitivity, selectivity, and long‐term stability for environmental and physiological sensors [37, 38]. Furthermore, in the context of optoelectronics, long operational lifetime, stretchability, color purity, and improved quantum efficiency are critical metrics for organic light‐emitting diodes (OLEDs) and electrochromic devices [39, 40, 41, 42], while low‐cost, solution‐processable, and green‐solvent‐compatible polymers are key to scaling next‐generation organic photovoltaics (OPVs) [43, 44, 45]. Achieving these multifunctional targets increasingly relies on the invention of new synthetic strategies and the rational design of unconventional polymer architectures [46, 47].

Given their unique structural and electronic characteristics, incorporating cross‐conjugated and broken‐conjugated building blocks into conjugated polymers may offer distinct advantages for these applications. Interestingly, some of these polymers even exhibit carrier mobilities comparable to those of their linear conjugated counterparts, opening new opportunities for materials design.

Polymers incorporating broken conjugation units have garnered growing interest for OLED applications due to their capacity to localize excitons and suppress non‐radiative decay pathways [48, 49, 50, 51, 52]. For instance, gridized nanopolymers based on fluorenyl moieties substituted at the 9‐position on sp^3^‐hybridized carbon centers form highly ordered three‐dimensional architectures with low conformational entropy and optoelectronic characteristics reminiscent of inorganic materials [53, 54]. These systems have demonstrated promising performance as efficient emitters with stable and tunable photophysical properties. Readers seeking further details are referred to a recent comprehensive review on this topic [54].

The present review focuses on cross‐conjugated polymer semiconductor systems, an unconventional but increasingly important class of materials. It summarizes recent advances in their structural design, fundamental properties, and emerging applications, categorized by key molecular features and performance criteria for different applications.

## 2 Arylamine‐Containing Cross‐Conjugated Polymers

**FIGURE 2:**
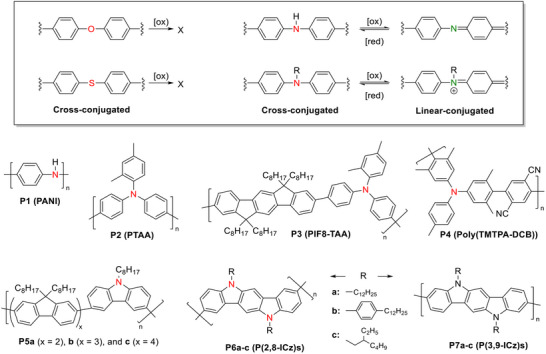
Susceptibility of aryl ethers, aryl thioethers, and arylamines toward oxidation and representative arylamine‐containing cross‐conjugated polymers. Cross‐conjugation sites are highlighted in red. Linearly conjugated polymers **P7a–c** are included as comparative references.

While cross‐conjugated polymers containing aryl ether or aryl thioether units, such as poly(phenylene oxide) (PPO) and poly(phenylene sulfide) (PPS), are typically insulating materials due to the inertness of their junction atoms (oxygen and sulfur, respectively), cross‐conjugated polymers with arylamine units form an important class of semiconducting and conductive materials for a wide range of applications. This is because the sp^3^‐hybridized nitrogen junction atom in an arylamine can be easily converted to sp^2^ hybridization, allowing it to act as a linear conjugation bridge that connects two neighboring conjugation systems, effectively extending π‐electron delocalization (FIGURE 2).

### 2.1 Polyaniline

**FIGURE 3:**
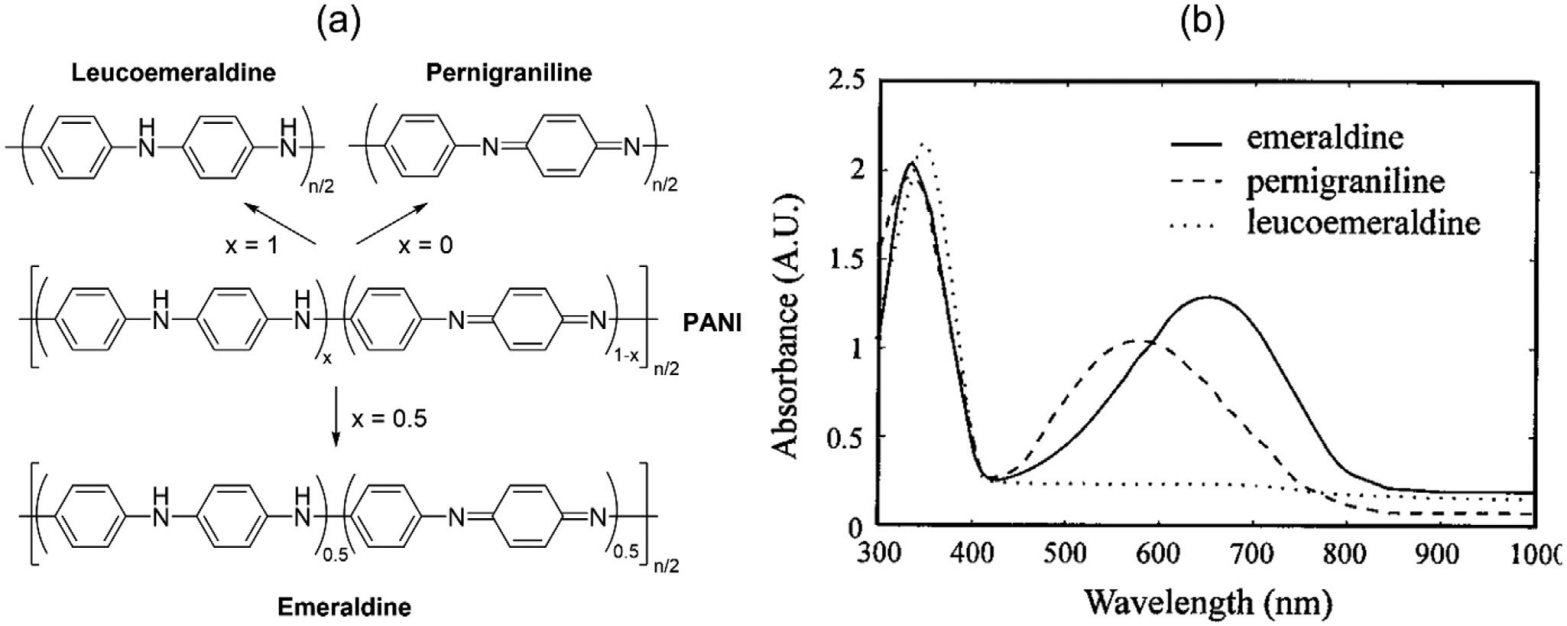
(a) Chemical structures and (b) UV–Vis absorption spectra of polyaniline (PANI, **P1**) in three distinct oxidation states: leucoemeraldine (fully reduced), emeraldine (semi‐oxidized), and pernigraniline (fully oxidized). Reproduced with permission [55]. Copyright 1997,Optica Publishing Group.

Polyaniline (PANI, **P1**, FIGURE 2) is arguably the simplest, earliest discovered, and most widely studied π‐conjugated polymer, with a broad range of applications including batteries, supercapacitors, and sensors [56, 57, 58, 59, 60, 61, 62, 63]. PANI can exist in three distinct oxidation states: leucoemeraldine (LEB), emeraldine (EB), and pernigraniline (PNB), as shown in FIGURE 3a. In its fully reduced LEB state, a cross‐conjugated form of PANI, exhibits a maximum absorption wavelength (*λ*_max_) at ≈350 nm (FIGURE 3b) [55], which is slightly redshifted compared to its monomer aniline (278 nm) [64] and the analogous small molecule *p*‐phenylenediamine (299 nm) [65]. In contrast to linearly conjugated polymers such as poly(3‐hexylthiophene), which display a redshift of over 200 nm compared to their monomers [66], the modest redshift seen in LEB suggests relatively weak electronic interactions between its repeat units.

In its fully oxidized PNB state, PANI adopts a linear conjugation along the backbone, leading to a significant redshift in *λ*_max_ to 543.5 nm. Interestingly, in the semi‐oxidized EB state [57], PANI exhibits an even further redshift in *λ*_max_ to 632.8 nm. Although emeraldine remains cross‐conjugated, its electronic transition involves excitation from the highest occupied molecular orbital (HOMO) localized on the electron‐rich benzenoid segments to the lowest unoccupied molecular orbital (LUMO) associated with the electron‐deficient quinoid segments. This intramolecular charge transfer (ICT) effectively reduces the HOMO‐LUMO bandgap [67].

PANI is nonconductive in all three neutral states (leucoemeraldine, emeraldine, and pernigraniline), but becomes conductive (up to 334 S cm⁻^1^) [68] when protonated or doped, forming an emeraldine salt. Carrier mobilities have been reported in the range of ≈10⁻^3^ to 10⁻^2^ cm^2^ V⁻^1^ s⁻^1^ for thin films [69, 70], and as high as 0.69 cm^2^ V⁻^1^ s⁻^1^ in nanofibers (Table 2) [71].

Numerous solution‐processable cross‐conjugated polymers incorporating N‐substituted diphenylamine units, such as poly[bis(4‐phenyl)(2,4,6‐trimethylphenyl)amine] (PTAA, **P2**) and poly(N,N‐bis‐4‐butylphenyl‐N,N‐bisphenyl)benzidine (poly‐TPD), have been developed over the years. These polymers exhibit moderate hole mobilities of up to 10⁻^4^–10⁻^2^ cm^2^ V⁻^1^ s⁻^2^ [72, 73] and are widely used as hole‐transporting materials (HTMs) in OLEDs [74] and perovskite solar cells [75, 76, 77, 78].

Incorporating linear conjugated comonomer units can significantly enhance charge transport. For instance, the copolymer of triphenylamine and indeno[1,2‐b]fluorene, PIF8‐TAA (**P3**), demonstrated an improved hole mobility of 0.04 cm^2^ V⁻^1^ s⁻^1^ [72]. Recently, donor‐acceptor (D‐A) polymers with electron‐withdrawing comonomer units have been integrated into poly(acrylamine) backbones to enable thermally activated delayed fluorescence (TADF) in OLEDs [79, 80]. Poly(TMTPA‐DCB) (**P4**) features triphenylamine (TPA) as the donor and dicyanobenzene (DCB) as the acceptor, with *ortho*‐methyl substituents enforcing a near‐perpendicular TPA‐DCB arrangement [79]. This design achieves an extremely low singlet‐triplet energy difference (Δ*E*_ST_) of 0.09 eV, enabling efficient TADF. OLEDs incorporating this polymer achieved a record‐high external quantum efficiency (EQE) of 24.0% (79.4 cd A^−1^, 75.0 lm W^−1^).

### 2.2 Carbazole‐Based Polymers

Carbazole (Cz), a fused diphenylamine with a planar structure, facilitates effective electron delocalization and is an essential building block in conjugated polymers due to its affordability, high environmental stability, and tunable optoelectronic properties [81, 82]. Similar to arylamine‐based polymers, carbazole‐containing polymers are widely studied as hole‐transporting materials in various organic electronic applications.

Polymers incorporating 3,6‐Cz units, such as poly(3,6‐Cz)s, are cross‐conjugated with the nitrogen atom at the 1‐position acting as a cross point. Compared to 2,7‐Cz‐based polymers, which are linearly conjugated, 3,6‐Cz‐based polymers generally exhibit larger bandgaps and lower carrier mobility [82, 83]. However, the *para*‐directing effect of the nitrogen atom in 2,7‐Cz renders the unblocked 3,6‐positions more susceptible to oxidation, making 2,7‐Cz‐containing polymers less stable and more challenging to synthesize [84, 85].

By introducing 3,6‐Cz units into polyfluorene backbones, fluorene‐carbazole copolymers (**P5a–c**, FIGURE 2) have demonstrated enhanced electrochemical stability and performance in OLED applications as blue light emitters and hole transport materials [86]. Despite the disrupted conjugation, energy transfer in these polymers remains efficient. Additionally, 3,6‐Cz‐containing polymers have been developed as efficient TADF emitters for OLEDs [87, 88, 89, 90].

Diphenylamine linked by an atom or a group such as oxygen [88], sulfur [91, 92], nitrogen, ketone, and methylene [93, 94, 95] form crucial building blocks for efficient polymeric TADF materials when incorporated through their cross‐conjugated positions—namely, *para* to nitrogen and/or nitrogen itself.

### 2.3 Indolo[3,2‐b]Carbazole‐Based Polymers

Indolo[3,2‐b]carbazole (ICz), a linearly extended fused‐ring structure, is highly coplanar, allowing π‐electron delocalization across the entire ICz unit. Consequently, ICz‐based small molecules exhibit high hole mobilities in OFETs [96, 97, 98]. Additionally, these molecules have weak absorption in the visible region, making them suitable for colorless, transparent electronics.

**FIGURE 4:**
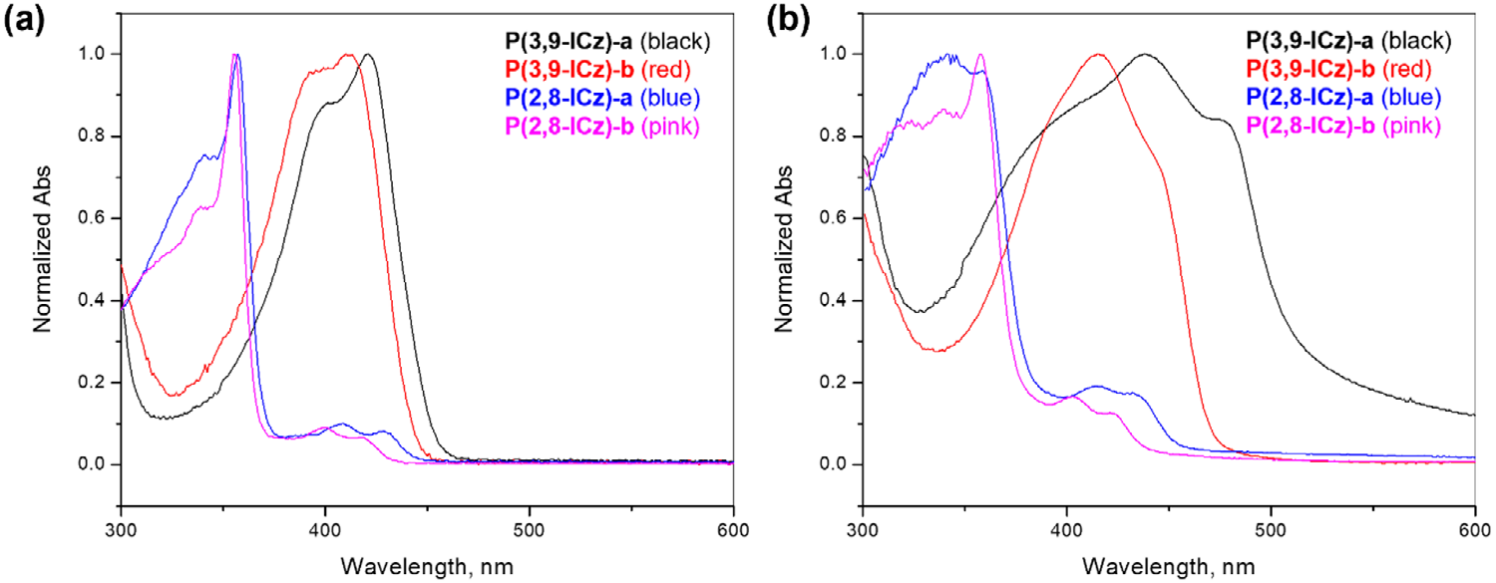
UV−vis absorption properties of P(2,8‐ICz)s (**P6a‐c**) P(3,9‐ICz)s (**P7a‐c**): (a) in THF solutions and (b) as thin films. Reproduced with permission [99]. Copyright 2006, ACS Publications.

Compared to their monomeric counterparts [97], polymers formed by linking ICz units at the 2,8‐positions (P(2,8‐ICz)s, **P6a,b**, FIGURE 2) show only slight redshifts and remain weakly absorbing in the visible range, as the 5,11‐nitrogen atoms disrupt backbone conjugation (FIGURE 4) [99]. Despite this disruption, P(2,8‐ICz) exhibits a hole mobility of up to 10^−2^ cm^2^ V^−1^ s^−1^ in OFETs. In contrast, 3,9‐ICz polymers (P(3,9‐ICz)s, **P7a,b**) show significantly enhanced absorption in the visible region due to effective linear conjugation along the backbone but lack field‐effect performance. Their poor solubility due to the rigid, linear backbone prevents the formation of sufficiently long polymer chains, hindering the development of high‐quality active‐layer films.

A direct comparison between a pair of soluble ICz polymers, P(2,8‐ICz) (**P6c**) and P(3,9‐ICz) (**P7c**), revealed that P(2,8‐ICz) exhibits higher conductivity (0.04 S cm⁻^1^) than P(3,9‐ICz) (0.02 S cm⁻^1^) [100], a trend also observed in their copolymers with bithiophene [101]. These findings suggest that P(2,8‐ICz) serves as a more efficient charge transport building block, likely due to the formation of a fully conjugated pathway via nitrogen atoms in the oxidized (doped) state.

## 3 Ketone‐Containing Polymers

A ketone carbonyl group is strongly electron‐withdrawing, which can form a D‐A‐D structure when connected to two electron‐donating conjugated units.

**FIGURE 5:**
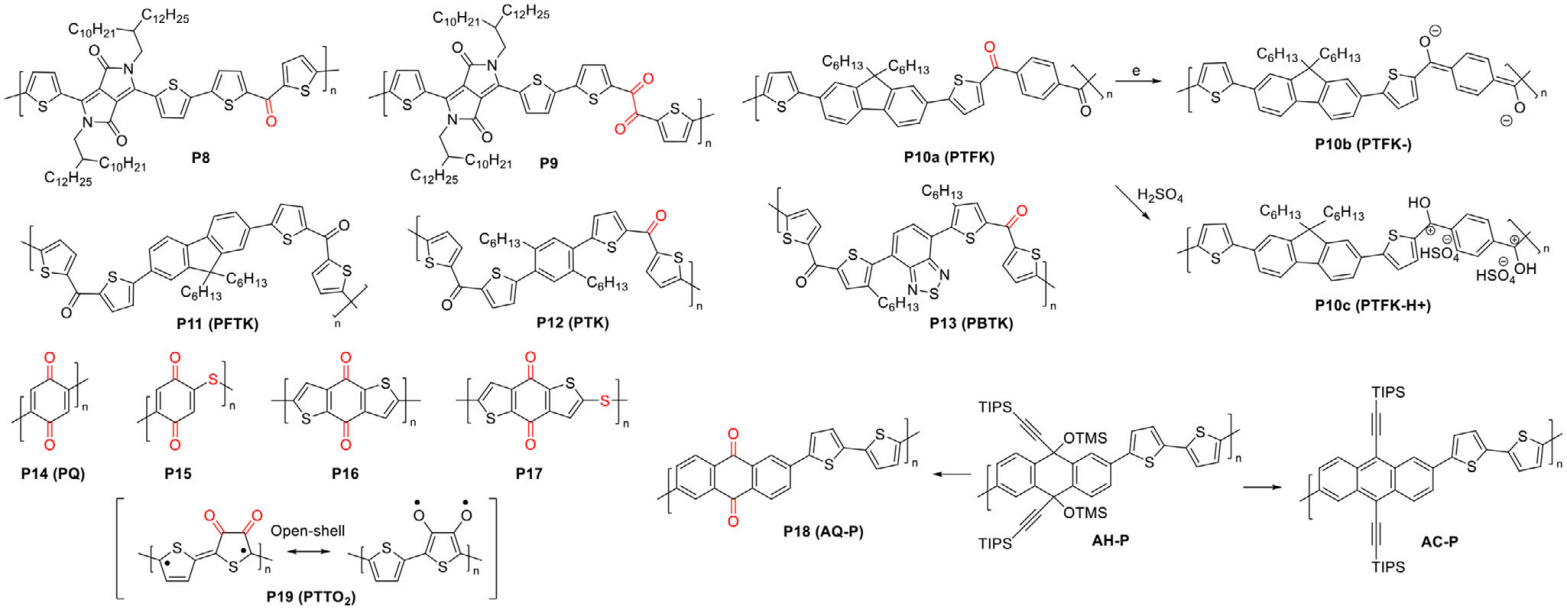
Representative ketone‐containing cross‐conjugated polymers. Cross‐conjugation sites are highlighted in red.

Zhang et al. reported a single ketone‐linked DPP copolymer (**P8**, figure 5) [102]. The polymer exhibited *λ*_max_ values of 730 nm in solution and 740 nm in film, which are blue‐shifted by 47 and 46 nm, respectively, compared to PDQT (a polymer without a ketone linkage) [103], indicating a shortened conjugation length. However, compared to small DPP molecules [104, 105] and an analogous DPP polymer with broken conjugation [106], **P8** showed noticeable redshifts, suggesting some degree of interaction between conjugated segments across the ketone group. **P8** achieved a moderate hole mobility of 0.12 cm^2^ V⁻^1^ s⁻^1^, significantly lower than that of PDQT (≈1–5.5 cm^2^ V⁻^1^ s⁻^1^) [103, 107], but much higher than polymers with broken conjugation (<0.032 cm^2^ V⁻^1^ s⁻^1^).

The same group also reported a DPP polymer, **P9**, incorporating a diketone group [108]. **P9** exhibited a *λ*_max_ of 756 nm in solution and 734 nm in thin film, red‐shifted and blue‐shifted, respectively, compared to **P8**. The polymer demonstrated an improved hole mobility of up to 0.22 cm^2^ V⁻^1^ s⁻^1^ in OFETs, attributed to enhanced coplanarity resulting from conformation locking between the diketone and adjacent thiophene units.

Voortman et al. proposed that the ketone groups in a cross‐conjugated polymer, PTFK (**P10a**), can be electrochemically reduced to form a conductive linearly conjugated polyionic (CPI) state [109]. The conductivity of the negatively charged reduced state (PTFK⁻, **P10b**) measured via cyclic voltammetry (CV) was on the order of 10⁻^3^ S cm⁻^1^. Interestingly, PTFK could also be chemically doped with H_2_SO_4_, forming a positively charged CPI state (PTFK⁻H⁺, **P10c**), which exhibited a dramatic redshift in *λ*_max_ (from 415 to 620 nm) and a reduction in *E*_g_ (from 2.6 to 1.7 eV) compared to neutral PTFK.

Ye et al. modified PTFK by replacing the phenylene spacer with bithiophene (BT) to form PFTK (**P11**) and further substituted the fluorene core with phenylene and 2,1,3‐benzothiadiazole, generating PTK (**P12**) and PBTK (**P13**), respectively [110]. Despite these structural variations, all polymers retained the ability to be doped by sulfuric acid, with protonation of carbonyl oxygen evidenced by significant redshifts in UV–Vis absorption compared to their neutral states.

These studies demonstrate that ketone‐based cross‐conjugated polymers (FIGURE 5) can be transformed into linearly conjugated states through electrochemical or chemical manipulation, offering a tunable approach to modifying electronic properties.

Leveraging its electron‐withdrawing properties and cross‐conjugation characteristics, the ketone linkage has been frequently incorporated with arylamine building blocks to develop TADF materials [95, 111, 112].

Small‐molecule and polymeric quinone compounds have garnered significant attention as organic electrode materials for rechargeable batteries, owing to their reversible redox behavior, high energy density, and, notably, their renewability for sustainable energy storage applications [113, 114, 115]. The simplest quinone polymer, poly(2,6‐quinone) (**P14**, PQ, FIGURE 5) [116, 117], features repeating 1,4‐quinone‐2,5‐diyl units, each having two ketone linkages. **P14** was first synthesized by Foos et al. in 1986 via a multi‐step process: electrochemical polymerization of dimethoxybenzene to form poly(1,4‐dimethoxybenzene) (PDMB), followed by HBr treatment to yield poly(1,4‐dihydroxybenzene) (PHQ), and subsequent electrochemical oxidation with bromine to produce **P14** [116]. When employed as the anode material with bromine and iodine as the catholytes, **P14**‐based batteries delivered first‐cycle discharge voltages/specific capacities of 2.80 V/≈140 mAh g^−1^ and 2.55 V/≈90 mAh g^−1^, respectively. Poly(benzoquinonyl sulfide) (**P15**, PBQS), which incorporates a sulfur linkage along with benzoquinone units in its backbone, was synthesized via the condensation of dichlorobenzoquinone with Li₂S, followed by oxidation using 2,3‐dichloro‐5,6‐dicyano‐1,4‐benzoquinone (DDQ) [118]. As a cathode material in lithium‐ion batteries (LIBs) and sodium‐ion batteries (NIBs), **P15** delivered high discharge voltages/specific capacities of 2.67 V/275 mAh g^−1^ and 2.08 V/268 mAh g^−1^, corresponding to impressive energy densities of 734 and 557 Wh kg^−1^, respectively. Analogous cross‐conjugated polymers (oligomers) based on benzo[1,2‐b:4,5‐b′]dithiophene‐4,8‐dione, **P16** (PBDTD) and **P17** (PBDTDS), were synthesized by Jing et al. [119], and investigated as cathode materials for lithium‐ion battery applications. Both polymers demonstrated high discharge voltages of ≈2.5 V versus Li/Li⁺ and specific capacities ≈200 mAh g⁻^1^, along with excellent cycling stability over 250 cycles. Notably, PBDTD exhibited improved electron transport in the reduced state due to the formation of a fully conjugated structure, whereas PBDTDS remained cross‐conjugated because of the presence of an electrochemically “inert” sulfur linkage.

Carlotti et al. reported a cross‐conjugated copolymer, AQ‐P (**P18**, figure 5), composed of anthraquinone (AQ) and bithiophene units, as well as a fully conjugated polymer, AC‐P, both synthesized from the precursor polymer AH‐P, which features non‐conjugated sp^2^ carbon linkages [120]. AH‐P, with its broken conjugation, exhibited a *λ*_max_ of ≈400 nm and an *E*_g_ of 2.8 eV, while AC‐P, possessing linear conjugation, showed a new broad absorption band ≈550 nm and a reduced *E*_g_ of 2.0 eV, indicative of significantly extended conjugation. Interestingly, the cross‐conjugated AQ‐P also displayed a small *E*_g_ of 2.0 eV, with a dominant absorption in the longer wavelength region compared to AC‐P. Although the authors did not discuss the origin of this unexpected observation, we believe it may be attributed to the formation of fully conjugated, biradical‐containing benzenoid resonance structures within the AQ units [121], which effectively enhances conjugation and lowers the bandgap of the quinone‐containing polymers. Despite this extended conjugation, **P18** showed relatively low hole and electron mobilities (≈10⁻^6^ cm^2^ V⁻^1^ s⁻^1^) in OFETs, comparable to those of the linearly conjugated AC‐P.

Another recent example is PTTO_2_ (**P19**), an alternating copolymer of thiophene‐[3,4]‐dione (TO_2_) and thiophene reported by Wei *et al.*, which also contains a biradical open‐shell structure and exhibits low‐energy absorption in the near‐IR region [122]. **P19** demonstrated an electrical conductivity of 10⁻^4^ S cm⁻^1^ in its undoped state. It also exhibited outstanding photothermal performance, reaching temperatures up to 274 °C under 1.2 W cm^−2^ irradiation.

## 4 Indigo‐Containing Polymers

Indigo (ID) is a cross‐conjugated building block, featuring a central vinylene bridge linking two indole moieties via one nitrogen and one ketone on each side. When ID is incorporated into a conjugated polymer through its 5,5’‐positions (5,5’‐ID), the two nitrogen atoms serve as crossing points, significantly disrupting conjugation (figure 1).

**FIGURE 6:**
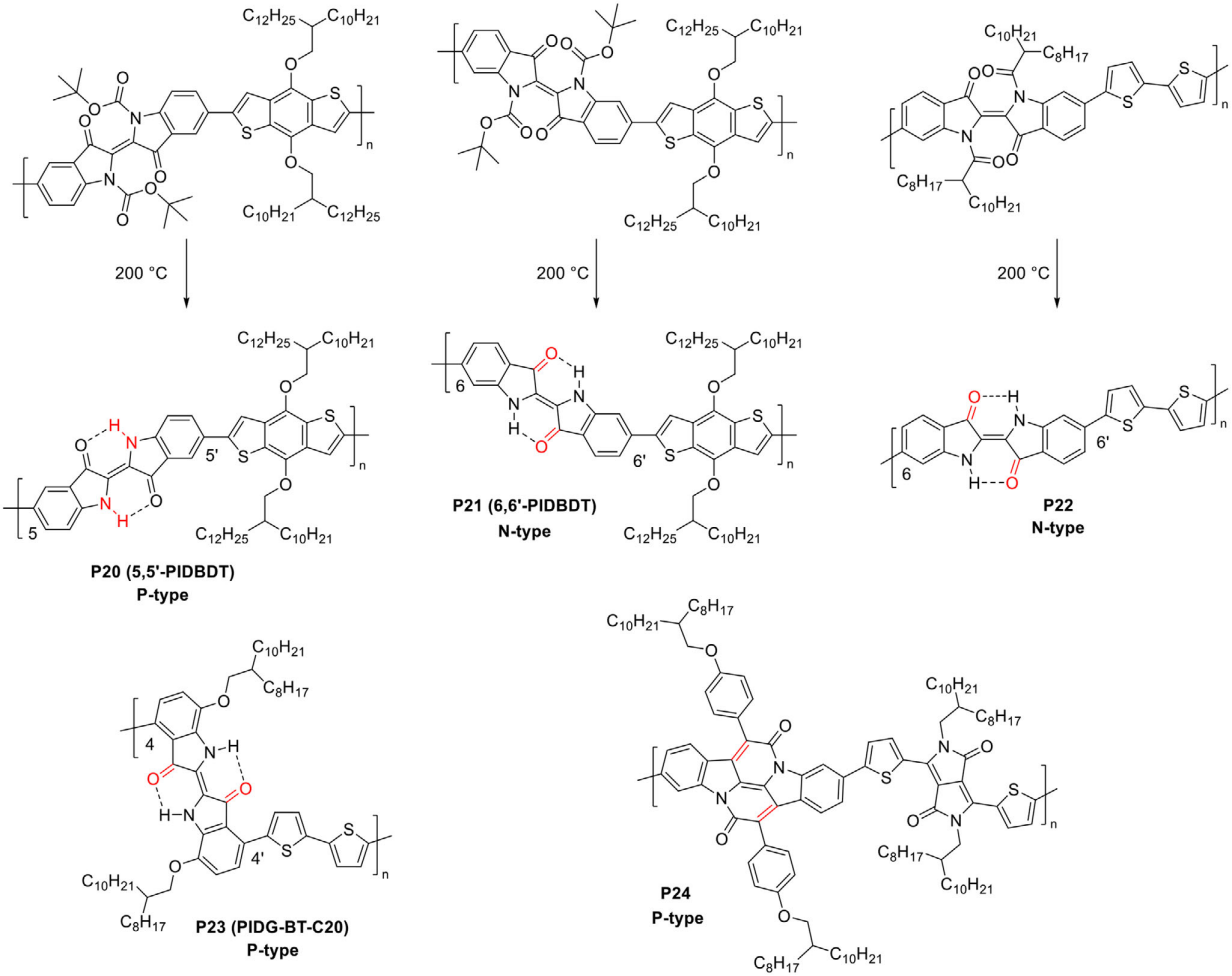
Representative ID‐containing cross‐conjugated polymers. Cross‐conjugation sites are highlighted in red.

Guo et al. synthesized a 5,5’‐ID‐based copolymer with benzodithiophene (BDT), **P20** (figure 6), incorporating thermally cleavable *t*‐butyl carbamate (*t*‐Boc) groups on nitrogen to enhance solubility [123]. After *t*‐Boc removal at 200 °C, the resulting polymer film exhibited a *λ*_max_ of 669 nm, red‐shifted compared to the ID monomer (5,5'‐dibromo‐[2,2'‐biindolinylidene]‐3,3'‐dione, *λ*_max_ = 621 nm), indicating extended backbone conjugation. **P20** displayed p‐type semiconductor characteristics in OFETs, achieving a hole mobility of 2.5 × 10⁻^4^ cm^2^ V⁻^1^ s⁻^1^. When ID is incorporated via its 6,6’‐positions, the two electron‐withdrawing ketone groups act as crossing points. The resulting isomeric polymer **P21** exhibited lower HOMO/LUMO energy levels (−5.8 eV/−4.2 eV) compared to its 5,5’‐counterpart **P20** (−5.3 eV/−3.6 eV). The lower LUMO facilitated stable electron transport, enabling electron mobility of 6 × 10⁻^3^ cm^2^ V⁻^1^ s⁻^1^ in n‐type OFETs [124]. Another 6,6’‐ID‐based polymer, **P22**, incorporating BT as a donor unit, also exhibited n‐type performance, achieving an electron mobility of 1.1 × 10⁻^3^ cm^2^ V⁻^1^ s⁻^1^ [125], highlighting the electron‐withdrawing effect of the ketone groups on charge transport.

Ngai et al. designed a copolymer, **P23**, where ID units were linked through their 4,4’‐positions to neighboring BT units, with long branched 2‐octyldodecyloxy side chains at the 7,7’‐positions to ensure solubility [126]. **P23** exhibited a *λ*_max_ of 758 nm and a narrow bandgap of 1.47 eV, representing a significant (≈90 nm) redshift compared to the ID monomer. This extended conjugation, compared to the 6,6’‐ID‐based **P22**, was likely due to the electron‐donating effect of alkoxy substituents at the 7,7’‐positions, which enhanced D‐A interactions between the indole moiety and ketone groups. The alkoxy groups also raised the HOMO level (–5.27 eV), significantly higher than **P22** (−5.78 eV), leading to enhanced p‐type OFET performance with a hole mobility of 0.028 cm^2^ V⁻^1^ s⁻^1^. Additionally, the N‐unsubstituted ID units in **P23** selectively interacted with fluoride ions via hydrogen bonding, enabling water‐gated OFET sensors with high fluoride sensitivity, achieving a limit of detection (LOD) of 0.40 mm.

Fallon et al. synthesized a bay‐annulated indigo derivative, indolonaphthyridine (IND), by reacting 6,6’‐dibromoindigo (Tyrian purple) with acryloyl chloride [127]. Unlike 6,6’‐ID, where ketone groups serve as crossing points, 6,6’‐IND features two vinyl groups as crossing points. A copolymer of 6,6’‐IND and DPP (**P24**) exhibited a *λ*_max_ of 727 nm in thin film, significantly redshifted compared to its monomers, indicating extended conjugation. In OFETs, **P24** demonstrated n‐type transistor performance with a maximum electron mobility of 0.10 cm^2^ V⁻^1^ s⁻^1^. Similar to 6,6’‐ID, 6,6’‐IND appears to promote electron transport, likely due to the electron‐withdrawing effect of the amide groups connected to the vinyl crossing points.

## 5 Amide‐Containing Polymers

**FIGURE 7:**
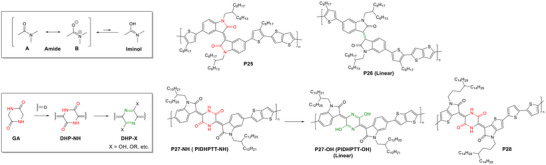
Tautomerization of amide building blocks, illustrated synthesis of the DHP building block, and representative IID and DHP‐containing cross‐conjugated polymers and their linearly conjugated counterparts. Cross‐conjugation sites are highlighted in red.

The amide group (Structure A, figure 7) can be considered a cross‐conjugated linkage, where the carbonyl acts as the crossing point, while the amino nitrogen acts as a conjugated unit through its lone pair. The amide group is known to exist in two states, amino‐carbonyl (A) and “amide resonance” (B) [128], as well as undergo the amide‐iminol tautomerism [129, 130]. Both B and the iminol form contain an unsaturated C=N bond, making them a conjugated linkage. According to a DFT simulation study by Kemnitz and Loewen, acetamide contains up to 28% of iminol form [128], while a study combining DFT simulation and mass spectroscopy conducted by Allegretti et al. on numerous compounds showed that some amides exist predominantly in the iminol form with tautomerism equilibrium constants of > 10^3^ [131].

### 5.1 Isoindigo‐Containing Polymers

Isoindigo (IID, figure 1) is an isomer of ID, differing in the positions of the ketone and nitrogen atom, which consists of two amide groups. Unlike ID, which has no linear conjugation pathway between two indole moieties, a linear conjugation can be achieved between the 6,6’‐positions. High‐performance linear conjugated polymers containing 6,6’‐IID have been developed in recent years, boosting high hole mobility of >5 cm^2^ V⁻^1^ s⁻^1^ in OTFTs [132, 133].

When IID is incorporated in the polymer through the 5,5’‐positions, the 5,5’‐IID building block is cross‐conjugated, where both amide groups become crossing groups, hindering extended conjugation.

Van Pruissen et al. investigated the impact of cross‐conjugation on the optoelectronic properties of two polymers, PcII‐TT (**P25**) and PII‐TT (**P26**) (figure 7), which contain 5,5’‐IID and 6,6’‐IID units, respectively [134]. Surprisingly, DFT simulations revealed that PcII‐TT has a slightly smaller *E*_g_ (1.71 eV, *E*_HOMO_/*E*_LUMO_ = −4.56 eV/−2.85 eV) than PII‐TT (1.77 eV, *E*_HOMO_/*E*_LUMO_ = −4.68 eV/−2.91 eV). These values align well with CV measurements, which reported *E*_g_/*E*_HOMO_/*E*_LUMO_ values of 1.69 eV/−5.59 eV/−3.90 eV for PcII‐TT and 1.71 eV/−5.62 eV/−3.91 eV for PII‐TT, respectively.

**FIGURE 8:**
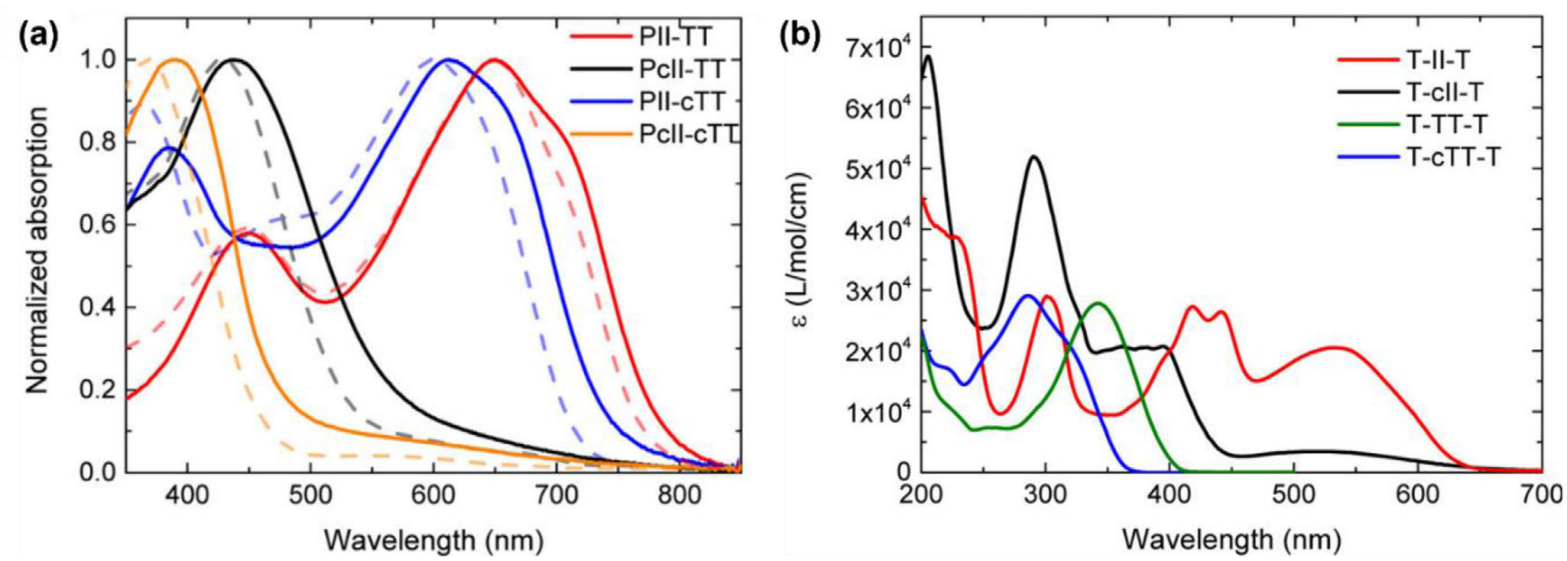
UV–vis absorption spectra of (a) PcII‐TT (**P25**) and PII‐TT (**P26**) in chloroform solution (dashed lines) and as thin films, and (b) their monomeric model compounds, T‐cII‐T and T‐II‐T, in heptane solution. Reproduced with permission [134]. Copyright 2015, ACS Publications.

However, their UV–Vis absorption spectra showed striking differences. PII‐TT exhibited a strong absorption peak at 650 nm in both solution and film, significantly redshifted compared to its monomeric model (T‐II‐T), indicating extended conjugation (figure 8). In contrast, PcII‐TT displayed a primary absorption peak at 429 nm in solution and 438 nm in film, closely matching the second‐strongest peak of its monomeric model (T‐IIc‐TT). This suggests that PcII‐TT has a much shorter effective conjugation length, likely due to the interruption caused by cross‐conjugated amide groups. Interestingly, PIIc‐TT exhibited an extended absorption tail reaching the near‐IR region, with an onset wavelength similar to that of PII‐TT. This extended tail contributes to its relatively narrow bandgap that was estimated from the onset wavelength.

To further explore this phenomenon, the authors conducted DFT simulations on the electronic transitions of cross‐conjugated T‐cII‐T. The HOMO–LUMO transitions for the first two excited states exhibited very low oscillator strengths (*f* = 0.03 and *f* = 0.00), with significant oscillator strength (*f* = 0.26) only appearing at the third transition (2.91 eV). In contrast, the linearly conjugated T‐II‐T showed a high oscillator strength for the first (*f* = 0.59 at 2.21 eV) and third (*f* = 0.52 at 2.84 eV) transitions. The lower oscillator strength in T‐cII‐T explains its reduced absorption at lower energy, despite having similar frontier orbital energy levels to its linear conjugated counterpart.

These findings further support the coexistence of linearly conjugated “amide resonance” and iminol forms, leading to transient linear conjugation along the polymer backbone.

### 5.2 Dihydropyrazine‐Containing Polymers

Recently, piperazine‐2,5‐dione (glycine anhydride, GA) has gained attention as a valuable and readily available precursor for synthesizing high‐performance π‐conjugated polymer semiconductors for diverse applications [136, 137, 138, 139, 140, 141, 142]. Its amide (lactam) form (DHP‐NH, figure 7) can be conveniently synthesized via Knoevenagel condensation of GA with formaldehyde or a ketone compound. To enable full conjugation along the polymer backbone, DHP‐NH must be converted into its iminol (or lactim) form (DHP‐X, where X = OH, alkoxy, etc.) through O‐substitution or other chemical modifications.

**FIGURE 9:**
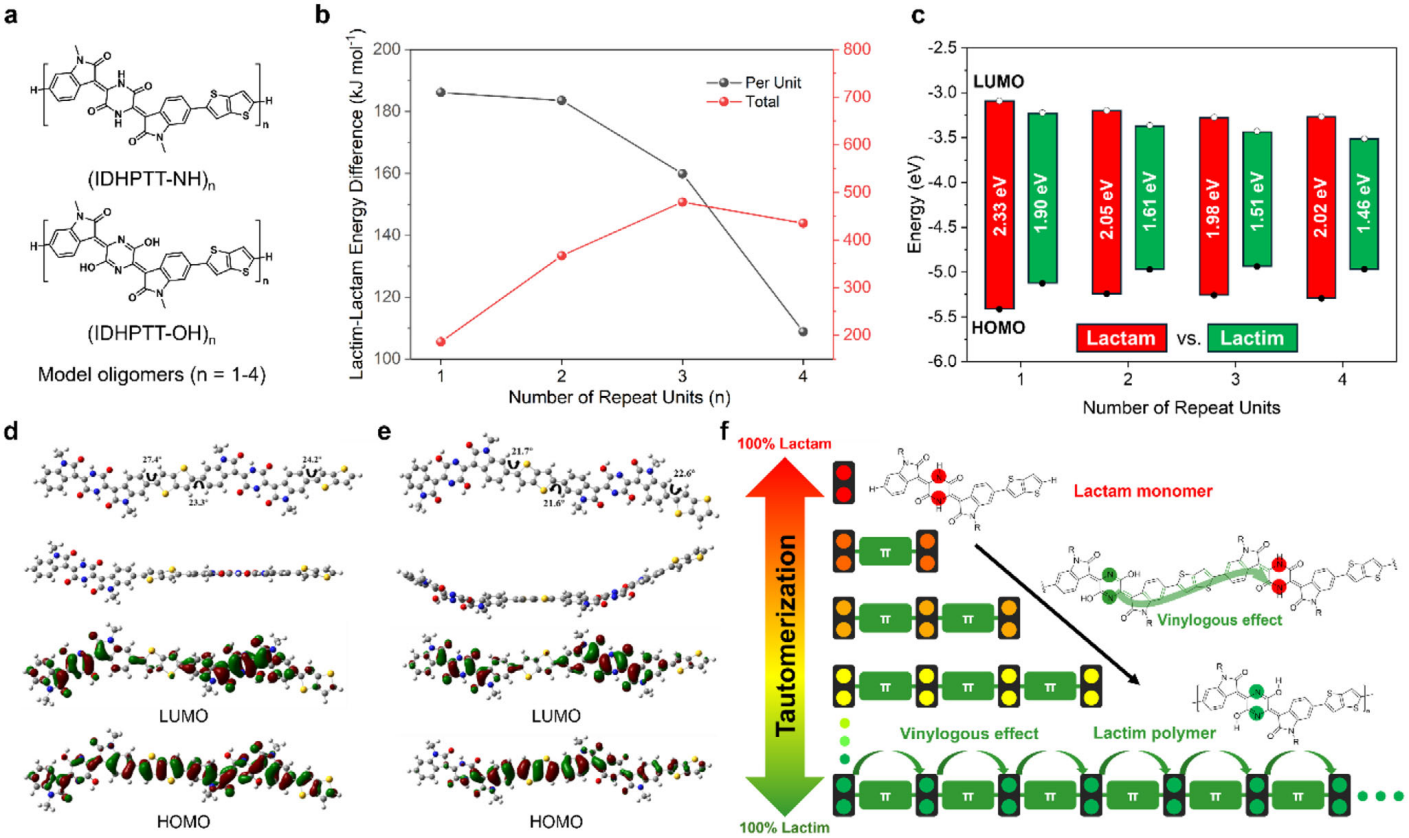
(a) Chemical structures of oligomers in lactam and lactim forms for PIDHPTT (**P27**) used in DFT simulations. (b) Energy differences between oligomers in their lactam and lactim forms obtained from DFT simulations. (c) Energy levels and bandgaps of oligomers obtained from DFT simulations. (d) Optimized geometries, HOMO, and LUMO of the lactam dimer, DIDHP‐NH. (e) Optimized geometries, HOMO, and LUMO of the lactim dimer, DIDHP‐OH. (f) Schematic illustration of the vinylogous effect promoting tautomerization from the predominant lactam monomer to the predominant lactim polymer with increasing chain length. Reproduced with permission [135]. Copyright 2024, John Wiley and Sons.

Given the promising fluoride sensing performance of the cross‐conjugated 4,4’‐ID‐based polymer [126], we strategically incorporated the unsubstituted DHP building block (DHP‐NH) into the polymer backbone to leverage the strong hydrogen‐bonding capability of its N‐H groups with fluoride ions (figure 9) [135]. Furthermore, the cross‐conjugated DHP‐NH unit may undergo a transition to a fully conjugated state under an applied gate bias, as previously proposed for single‐molecule transistors based on cross‐conjugated systems, potentially enabling conductance enhancements of up to eight orders of magnitude [143]. Specifically, a DHP‐NH‐containing monomer, IDHP, flanked by two oxindole moieties, was copolymerized with thienothiophene (TT) to yield the polymer PIDHPTT (**P27‐NH**). Surprisingly, UV–Vis spectra of PIDHPTT exhibited significant redshifts, displaying two strong low‐energy absorption peaks at 654 and 721 nm in solution, compared to its monomer IDHP‐Br, which had a much shorter *λ*_max_ of 417 nm. The polymer film showed a narrow bandgap of 1.57 eV, calculated from its onset wavelength, indicating highly efficient conjugation along the backbone.

More intriguingly, Fourier transform infrared (FTIR) analysis showed that while the IDHP‐Br monomer exhibited characteristic N‐H stretching (*υ* = 3100 cm^−1^) and N‐H bending (*υ* = 1591 cm^−1^) vibrations, these signals were absent in PIDHPTT. Instead, the polymer displayed distinct O─H stretching (*υ* = 3660 cm⁻^1^) and C‐OH stretching peaks, suggesting that the DHP units predominantly exist in the lactim (DHP‐OH) form (**P27‐OH**). DFT simulations of oligomers revealed that the lactim configuration becomes increasingly stabilized as the number of repeat units grows, indicating that the lactam‐to‐lactim conversion in DHP is influenced by the conjugation environment. A series of oligomers were synthesized and analyzed via FTIR and NMR spectroscopy, experimentally confirming progressive conversion from DHP‐NH to DHP‐OH with increasing chain length. These findings suggest that the presence of a neighboring DHP‐OH unit facilitates the lactam‐to‐lactim conversion, a vinylogous effect previously observed only in small molecules. This study represents the first report of such a long‐range vinylogous effect in a polymer.

**P27‐OH** exhibited ambipolar charge transport in organic field‐effect transistors (OFETs), achieving balanced hole and electron mobilities of 0.0245 and 0.0349 cm^2^ V^−1^ s^−1^, respectively. This balanced transport is attributed to its favorable frontier orbital levels (*E*_HOMO_ = −5.58 eV, *E*_LUMO_ = −3.71 eV), which support both hole and electron conduction.

Water‐gated OFETs with **P27‐OH** as the active and sensing layer demonstrated exceptional selectivity for fluoride ions over other halides, achieving a remarkably low limit of detection (LOD) of 0.28 *µ* m. The high fluoride sensitivity was attributed to the strong interaction between fluoride ions and the O‐H groups in DHP, as corroborated by computer simulations, UV–vis measurements, and electrochemical impedance spectroscopy (EIS).

Wang et al. reported a DHP‐based building block incorporating thieno‐oxindole instead of oxindole [144]. The resulting polymer, **P28**, synthesized via copolymerization with bithiophene, exhibited a hole mobility of 1.58 cm^2^ V^−1^ s^−1^, the highest among previously reported cross‐conjugated polymers to date. This exceptional charge transport performance is likely attributed to the transition from cross‐conjugation to linear conjugation via lactam‐to‐lactim tautomerization, as observed in **P27** [126], which enhances electronic delocalization and charge carrier mobility.

## 6 Thieno[2,3‐b]Thiophene‐Containing Polymers

**FIGURE 10:**
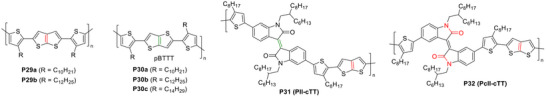
Representative cross‐conjugated polymers incorporating the thieno[2,3‐b]thiophene (cTT) building block. Cross‐conjugation sites are highlighted in red. Linearly conjugated polymers **P30a–c** are included as comparative examples.

While thieno[3,2‐b]thiophene (TT) serves as a fully conjugated building block when incorporated into a polymer backbone through the 2,5‐positions, its isomer, thieno[2,3‐b]thiophene (cTT), introduces cross‐conjugation. Both TT and cTT have been widely employed as key structural units in the design of conjugated polymers [145, 146]. Heeney et al. utilized cTT to synthesize cross‐conjugated copolymers, **P29a** and **P29b** (figure 10), by copolymerizing it with bithiophene units bearing different alkyl side chains [147]. These polymers exhibited p‐type semiconducting behavior in organic field‐effect transistors (OFETs), achieving hole mobilities of 0.15 and 0.12 cm^2^ V⁻^1^ s⁻^1^, respectively—outperforming the widely studied fully conjugated regioregular poly(3‐hexylthiophne) (P3HT), which typically shows mobilities ≈0.1 cm^2^ V^−1^ s^−1^. However, their performance lagged behind that of analogous fully conjugated polymers, **P30a** and **P30b**, which incorporate linearly conjugated TT and exhibited hole mobilities of up to 0.30 cm^2^ V^−1^ s^−1^ [148]. Notably, **P30c** (pBTTT‐C14), featuring longer tetradecyl side chains, achieved even higher mobility values of up to 0.72 cm^2^ V⁻^1^ s⁻^1^ and has since become one of the most widely studied p‐type polymer semiconductors for various applications.

Van Pruissen further explored the effect of cTT on the properties of polymers by synthesizing a cross‐conjugated polymer, PII‐cTT (**P31**), composed of isoindigo (II) and cTT units [134]. Compared to its fully conjugated counterpart, PII‐TT (**P26**, figure 7), **P31** exhibited a blue‐shifted UV–Vis absorption spectrum; however, the absorption remained significantly red‐shifted relative to its constituent monomers, T‐II‐T and T‐cTT‐T (FIGURE 8), indicating some extension of conjugation through the cTT units. In contrast, PcII‐cTT (**P32**), which incorporates both cross‐conjugated cII and cTT units, exhibited a pronounced blue shift of over 50 nm compared to PcII‐TT (**P25**), which contains cross‐conjugated cII but linearly conjugated TT. The absorption spectrum of PcII‐cTT closely resembled those of its monomers, T‐cII‐T and T‐cTT‐T, indicating that conjugation was almost completely disrupted due to the presence of two cross‐conjugated building blocks in the repeating unit.

## 7 2D Cross‐Conjugated Polymers

**FIGURE 11:**
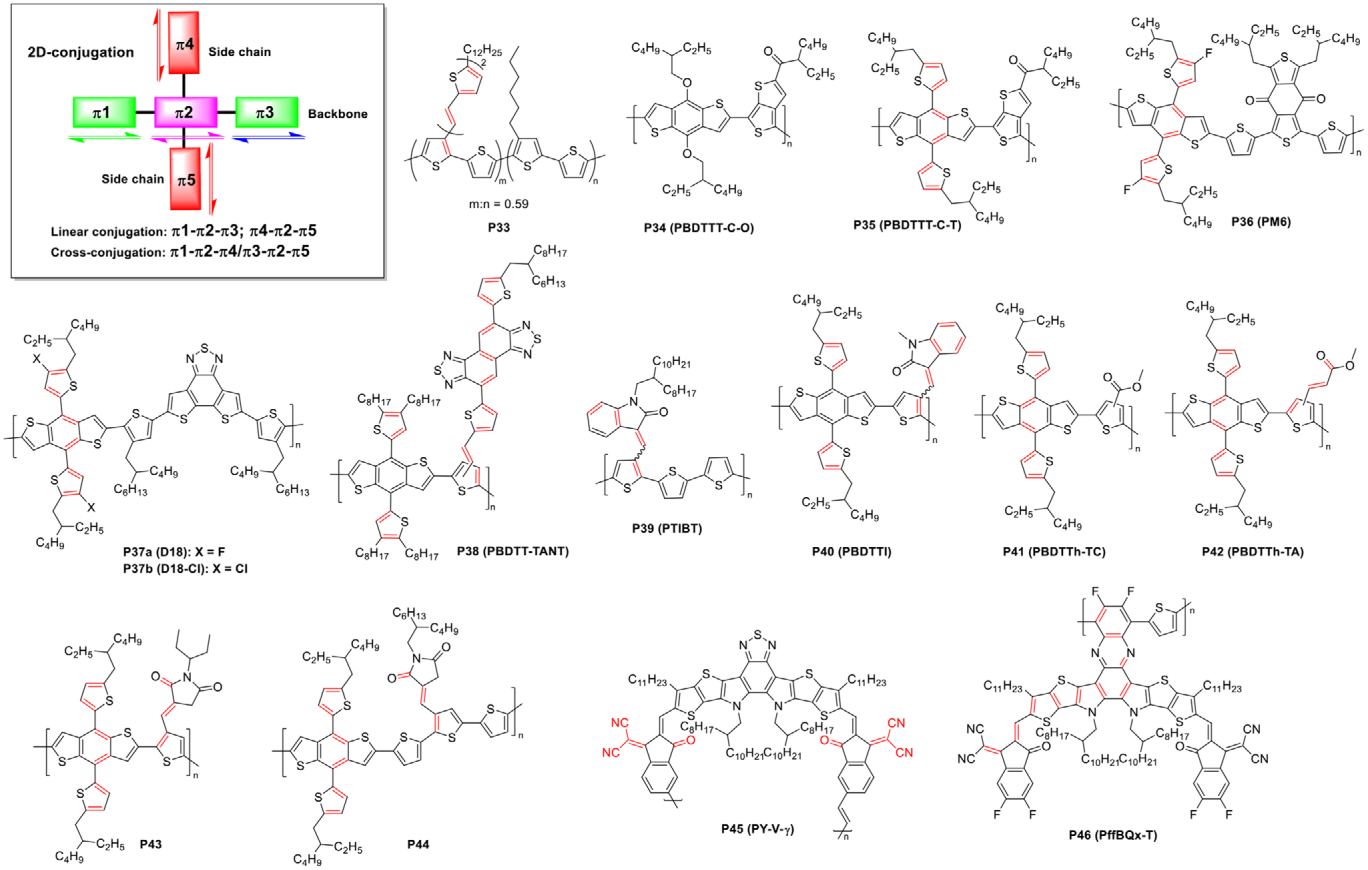
Representative 2D cross‐conjugated polymers. Cross‐conjugation systems or sites are highlighted in red. The linearly conjugated polymer **P34** and one‐dimensional cross‐conjugated polymer **P45** are included as comparative examples.

Conjugated polymers feature 2D linear conjugation through both the main backbone and side chains, but a cross‐conjugation relationship between them, offer unique opportunities for modulating electronic, optical, and electrochemical properties (FIGURE 11). In 2006, Hou et al. reported a 2D‐conjugated polythiophene (**P33**) with bi(thienylenevinylene) (biTV) side chains for use in organic photovoltaics (OPVs) [149]. Compared to P3HT, which lacks conjugated side chains, **P33** exhibited a stronger and broader absorption profile from 350 to 480 nm and showed complete exciton energy transfer from the side chains to the backbone. These features contributed to a 35% improvement in power conversion efficiency (PCE), reaching 3.18% compared to devices based on P3HT:PC_61_BM.

The same group later extended this design strategy to benzo[1,2‐b:4,5‐b′]dithiophene (BDT) by replacing non‐conjugated alkoxy groups with conjugated alkylthienyl groups [150]. The resulting polymer (**P35**) displayed a high space charge limited current (SCLC) hole mobility of 0.27 cm^2^ V^−1^ s^−1^, over three orders of magnitude higher than the alkoxyl‐substituted counterpart **P34** (5.53 × 10^−4^ cm^2^ V^−1^ s^−1^). OPVs based on **P35**:PC_71_BM achieved high PCE up to 7.6%, a notable increase compared to the **P34**:PC_71_BM‐based devices (6.4%). This pioneering work established alkylthienyl‐substituted BDTs as a leading class of polymer donor building blocks [151, 152, 153, 154, 155], including the current state‐of‐the‐art donor materials PM6 (**P36**) [156, 157], D18 (**P37a**) [158, 159], and D18‐Cl (**P37b**) [160, 161].

Efforts have also focused on incorporating 2D‐conjugated acceptor units into polymer donors. Xu et al. developed a polymer (**P** **38**) with large, planar, electron‐deficient naphthobisthiadiazole (NT) side chains [162]. These side chains helped lower the HOMO energy level and improve crystallinity and hole mobility, contributing to enhanced open‐circuit voltage (*V*_OC_), short‐circuit current density (*J*_SC_), fill factor (FF), and overall PCE in **P38**:PC_71_BM devices.

Li et al. proposed a donor‐backbone/acceptor‐side‐chain design by attaching extended conjugated electron‐withdrawing side chains to electron‐donating backbones, resulting in wide‐bandgap polymer donors suitable for pairing with small‐bandgap non‐fullerene acceptors (NFAs) [163, 164, 165, 166]. Unlike conventional D–A backbones, this architecture offers several advantages: 1) exciton polarization by the acceptor side chains reduces binding energy; 2) the side chains minimally disrupt backbone conjugation, maintaining a wide bandgap; 3) the donor‐only backbone supports efficient hole transport; and 4) greater synthetic flexibility and potentially lower costs [163].

For example, **P39** (PTIBT), featuring a polythiophene backbone and indolin‐2‐one side chains, was synthesized via a three‐step route with low synthetic complexity (SC = 23.5%) [163]. The polymer exhibited a high dielectric constant (7.70) and a hole mobility of 1.81 × 10^−2^ cm^2^ V^−1^ s^−1^ in OFETs. However, its OPV performance was limited (PCE = 5.72%) due to a high HOMO energy level and edge‐on chain packing, resulting in poor vertical mobility (1.79 × 10^−4^ cm^2^ V^−1^ s^−1^). To overcome these drawbacks, **P40** was designed by incorporating BDT comonomers, which promote face‐on packing and lower the HOMO level [164]. The resulting polymer achieved vertical mobility of 4.4 × 10^−3^ cm^2^ V^−1^ s^−1^ and an improved PCE of 8%.

Yuan et al. developed 2D‐conjugated polymer **P42** using conjugated acrylate side chains, resulting in an increased dipole moment (4.82 D), a higher dielectric constant (3.78), and reduced exciton binding energy (0.415 eV) compared to the carboxylate analog **P41** [165]. **P42** also showed superior hole mobility (1.11 × 10^−4^ cm^2^ V^−1^ s^−1^) compared to **P41** (4.39 × 10^−5^ cm^2^ V^−1^ s^−1^), yielding better OPV device performance.

Extending this strategy, Yuan et al. developed **P43** and **P44** using vinyl‐bridged succinimide side chains [166]. While **P43**:Y6‐based devices demonstrated low energy loss (*E*_loss_ = 0.44 eV), their performance was constrained by low hole mobility (1.52 × 10^−4^ cm^2^ V^−1^ s^−1^). Incorporating thiophene spacers and fluorinated BDT units in **P44** improved vertical mobility (1.04 × 10^−3^ cm^2^ V^−1^ s^−1^), resulting in OPVs with a high PCE of 13.21% and *J*_SC_ of 26.83 mA cm^−2^, significantly outperforming **P43** (PCE = 8.51%; *J*_SC_ = 19.36 mA cm^−2^).

Recently, Yan et al. reported a novel 2D‐conjugated polymer acceptor, **P46** (PffBQx‐T), and compared it to a linear, cross‐conjugated analog, **P45** [167]. The double‐decker architecture of **P46** facilitated tight intramolecular packing between its pendent conjugated side chains, achieving a short π–π stacking distance of 3.62 Å, significantly shorter than the 3.76 Å intermolecular stacking observed in **P45**. This compact packing enabled a more efficient intramolecular charge transport pathway relative to its linear counterpart. As a result, PM6:**P46** all‐polymer solar cells exhibited enhanced *V*_OC_ and FF, yielding a high PCE of 17.0%, comparable to that of PM6:**P45**‐based devices (17.3%). Notably, ternary PM6:**P45**:**P46** devices achieved a record PCE of 18.7%, benefiting from improved charge transport, reduced energy loss, and enhanced operational stability.

In summary, 2D‐conjugated polymer designs enable enhanced light absorption and charge transport, while reducing exciton binding energy. These attributes make them powerful candidates for next‐generation OPVs and highlight the importance of precise conjugated side‐chain engineering in conjugated polymer semiconductors.

**TABLE 2:** Summary of key properties and potential applications of polymers discussed in this review.

No.	Short Name	*E*_HOMO_/*E*_LUMO_/*E*_g_^opt^ (eV)^a^ ^)^	*λ*_max_ (nm)	Mobility (cm^2^ V^−1^ s^−1^)	Application/Performance^b)^
**P1** [67]	PANI	LEB:–/–/3.94 EB:–/–/1.5	315 (LEB) 590 (EB)	∼10^−3^ to 10^−2^ (film) [69, 70] 0.69 (nanofiber) [71]	Conductive polymer / σ = 334 S cm^−1^ (ref. [68]); Batteries, supercapacitors, and sensors [57, 58, 59, 60, 61, 62, 63]
**P2** [72]	PTAA	−5.2 /–/–	—	OFET: ≈4 × 10^−3^ (hole)	Active layer in OFETs; HTM for perovskite solar cells / PCE = 18% [75]
**P3** [72]	PIF8‐TAA	−5.5/–/–	—	OFET: ≈0.04 (hole)	Active layer in OFETs
**P4** [79]	Poly(TMTPA‐DCB)	−5.26/−2.92/2.34	≈300	—	Green TADF emitter for OLEDs / EQE = 24.0%
**P5** [86]	P5a (P(F2‐alt‐Cz)) P5b (P(F3‐alt‐Cz)) P5c (P(F4‐alt‐Cz))	‒5.34/−2.04/3.30 (P(F2‐alt‐Cz)) ‒5.39/−2.17/3.22 (P(F3‐alt‐Cz)) ‒5.42/−2.21/3.21 (P(F4‐alt‐Cz))	367 (P(F2‐alt‐Cz)) 371 (P(F3‐alt‐Cz)) 376 (P(F4‐alt‐Cz))	—	Blue OLEDs / Luminance = 350 cd/m^2^ (P(F3‐*alt*‐Cz))
**P6** [99, 100]	P(2,8‐ICz)s	−5.1 to –5.2/–/–	425/422/425	OFET: ≈10^−5^ to 10^−2^ (hole)	Active layer in OFETs; Conductive polymer / σ = 0.04 S cm^−1^
**P7** [99, 100]	P(3,9‐ICz)s	—	477/444/—	—	Conductive polymer / σ = 0.002 S cm^−1^
**P8** [102]	PDMO‐S	−5.57/−3.58/1.38	740	OFET: 0.12 (hole)	Active layer in OFETs
**P9** [108]	PDTO‐C1	−5.53/−3.64/1.38	734	OFET: 0.22 (hole)	Active layer in OFETs
**P10** [109]	P10a (PTFK) P10b (PTFK^–^) P10c (PTFK^–^H^+^)	−/−/2.6 (PTFK) −/−/− (PTFK^−^) −/−/1.7 (PTFK^−^H^+^)	415 (PTFK) – (PTFK^–^) 620 (PTFK^–^H^+^)	PTFK: 1.3 × 10^−8^ (electron); 5.2 × 10^−7^ (hole) (zero‐field mobility)	Yellow emitter for OLEDs / 570 nm
**P11** [110]	PFTK	−5.30/−2.94/2.30	450	—	—
**P12** [110]	PTK	−5.50/−2.99/2.51	420	—	—
**P13** [110]	PBTK	−5.34/−3.04/2.36	445	—	—
**P14** [116]	PQ	—	—	—	Br_2_ batteries / 2.80 V and ≈140 mAh g^−1^; I_2_ batteries / 2.55 V and ∼90 mAh g^−1^
**P15** [118]	PBQS	—	—	—	LIBs/2.67 V and 275 mAh g^−1^; NIBs/2.08 V and 268 mAh g^−1^
**P16** [119]	PBDTD	—	—	—	LIBs/2.5 V and 200 mAh g^−1^
**P17** [119]	PBDTDS	—	—	—	LIBs/2.5 V and 200 mAh g^−1^
**P18** [120]	AQ‐P	−5.34/−3.04/2.0	∼480	2.0 × 10^−6^ (hole) 3.0 × 10^−6^ (electron)	Active layer in OFETs Electrochromic devices
**P19** [122]	PTTO_2_	−5.14/–/0.82	—	—	Photothermal material / σ = 3.11 × 10^−4^ S cm^−1^
**P20** [123]	5,5’‐PIDBDT	−5.3/−3.6/1.7	669	OFET: 2.5 × 10^−4^ (hole)	Active layer in OFETs
**P21** [124]	6,6’‐PIDBDT	−5.8/−4.2/1.6	641	OFET: 6 × 10^−3^ (electron)	Active layer in OFETs
**P22** [125]	PIIDBT‐20	−5.70/−3.70/2.00	701	OFET: 1.1 × 10^−4^ (electron)	Active layer in OFETs
**P23** [126]	PIDG‐BT‐C20	−5.27/−3.80/1.47	758	OFET: 0.028 (hole)	Active layer in OFETs; Active layer in water‐gated OFET fluoride sensor / LOD = 0.40 mM
**P24** [127]	XINDB‐DPPT	−5.29/−3.75/1.54	727	OFET: 0.10 (electron)	The active layer in OFETs
**P25** [134]	PcII‐TT	−5.59/−3.90/1.69	438	—	—
**P26** [134]	PII‐TT	−5.62/−3.91/1.71	650	—	—
**P27** [135]	PIDHPTT	−5.58/−3.71/1.87	718	OFET: 0.0245 (hole); 0.0349 (electron)	Active layer in OFETs; Active layer in water‐gated OFET fluoride sensor / LOD = 0.28 µM
**P28** [144]	PTIDP2T	−4.95/−3.72/1.23	887	OFET: 1.58 (hole)	Active layer in OFETs
**P29a** [147]	—	−5.30/–/–	470	OFET: 0.15 (hole)	Active layer in OFETs
**P29b** [147]	—	−5.30/–/–	472	OFET: 0.12 (hole)	Active layer in OFETs
**P30a** [148]	pBTTT‐C10	−5.10 /–/ –	547	OFET: 0.30 (hole)	Active layer in OFETs
**P30b** [148]	pBTTT‐C12	−5.10/–/–	547	OFET: 0.30 (hole)	Active layer in OFETs
**P30c** [148]	pBTTT‐C14	−5.10/−3.19/1.91 [168]	547 [148] / 559 [168]	OFET: 0.72 (hole)	Active layer in OFETs
**P31** [134]	PII‐cTT	−5.83/−3.94/1.52	364, 613	—	—
**P32** [134]	PcII‐cTT	−5.77/–3.99/1.56	370	—	—
**P33** [149]	—	−4.93/−2.96/1.82	452; 540	—	OPVs / PCE = 3.18% (**P33**:PC_61_BM)
**P34** [150]	PBDTTT‐C‐O	−5.07/−3.47/1.60	—	OFET: 5.53 × 10^−4^ (hole)	OPVs / PCE = 6.34% (**P34**: PC_71_BM)
**P35** [150]	PBDTTT‐C‐T	−5.11/−3.53/1.58	—	OFET: 0.27 (hole)	OPVs / PCE = 7.59% (**P35**: PC_71_BM)
**P36** [156]	PM6	−5.45/−3.65/1.80	550	SCLC: 2.77 × 10^−4^ [157]	OPVs / PCE = 15.7% (**P36**:Y6) [157]
**P37a** [158]	D18	−5.51/−2.77/1.98	555; 581	SCLC: 1.59 × 10^−3^	OPVs / PCE = 18.22% (**P37a**:Y6)
**P37b** [160]	D18‐Cl	−5.56/−2.78/1.99	574	SCLC: 1.00 **×** 10^−3^	OPVs / PCE = 18.13% (**P37b**:N3) [160]; 20.82% (**P37b**:N3:AT‐β_2_O) [161]
**P38** [162]	PBDTT‐TANT	−5.37/−3.39/1.98	516	SCLC: 2.93 × 10^−4^	OPVs / PCE = 8.04% (**P38**: PC_71_BM)
**P39** [163]	PTIBT	−5.41/−3.61/1.80	576	OFET: 1.81 × 10^−2^ (hole) SCLC: 1.79 × 10^−4^	*ε*_r_ = 7.70 OPVs / PCE = 5.72% (**P39**:ITIC)
**P40** [164]	PBDTTI	−5.59/−3.69/1.91	460; 566; 649	OFET: 4.4 × 10^−3^ (hole)	OPVs / PCE = 8.00% (**P40**:ITIC)
**P41** [165]	PBDT‐TC	−5.49/−3.49/2.00	534; 563 (sh)	SCLC: 7.59 × 10^−5^	OPVs / PCE = 9.68% (**P41**:ITIC)
**P42** [165]	PBDT‐TA	−5.46/−3.44/2.02	541	SCLC: 2.00 × 10^−4^	OPVs / PCE = 10.47% (**P42**:ITIC)
**P43** [166]		−5.53/−3.53/2.00	556	SCLC: 1.52 × 10^−4^	OPVs / PCE = 8.51% (**P43**:Y6); *E*_loss_ = 0.44 eV
**P44** [166]	—	−5.51/−3.57/1.94	547	SCLC: 1.04 × 10^−3^	OPVs / PCE = 13.21% (**P44**:Y6)
**P45** [167]	PY‐V‐*γ*	−5.64/−3.76/1.41	827	—	OPVs / PCE = 17.3% (PM6:**P45**)
**P46** [167]	PffBQx‐T	−5.72/−3.77/1.54	809	—	OPVs / PCE = 17.0% (PM6:**P46**); 18.7% (PM6:**P45**:**P46**)

^a)^ *E*_g_^opt^: Optical bandgap determined from the onset absorption wavelength.

^b)^ For OFET applications, performance data are provided in the “Mobility” column.

## 8 Conclusion and Future Directions

Cross‐conjugated polymers present an unconventional yet promising avenue for designing semiconducting materials with tunable and multifunctional properties. Although their interrupted π‐conjugation pathways often lead to lower charge carrier mobilities compared to fully linear systems, this limitation is offset by unique advantages—such as enhanced light‐emission efficiency, reversible conjugation switching, reversible electrochemical processes, and the formation of biradical or zwitterionic resonance structures under specific stimuli. These characteristics make cross‐conjugated polymers particularly appealing for applications beyond conventional OFETs, including sensors, electrochromic devices, TADF‐based OLEDs, and organic batteries.

Recent advances have shown that some cross‐conjugated polymers can approach, or even rival, the performance of linearly conjugated counterparts—especially when capable of transitioning into a fully conjugated state through doping or tautomerization. Strategies involving responsive linkers (e.g., arylamines, ketones, amides) or exploiting tautomerism (e.g., in DHP‐ and amide‐containing backbones) have enabled the development of polymers with enhanced conjugation, stability, and functional responsiveness. Notably, materials such as PIDHPTT and PTTO₂ have demonstrated promising capabilities in chemical sensing and photothermal conversion, respectively.

Looking forward, several research directions warrant further exploration:

- Unexplored Cross‐Conjugated Building Blocks: Historically viewed as inferior, cross‐conjugated units have received limited attention. Systematic exploration of existing and newly designed cross‐conjugated building blocks could yield materials with unprecedented properties for diverse applications.
- Conjugation Switching Mechanisms: A deeper understanding of reversible conjugation transitions—triggered by electrical, chemical, or environmental stimuli—may unlock new device functionalities.
- High‐Mobility Architectures: Rational backbone design and side‐chain engineering could enhance charge transport while preserving the benefits of cross‐conjugation.
- Emerging Applications: Cross‐conjugated systems hold untapped potential in areas such as electrochemical energy storage, bioelectronics, and multifunctional sensing.
- Theoretical and Computational Tools: Advanced modeling techniques, including DFT, will be essential for elucidating charge transport mechanisms and guiding the rational design of next‐generation materials.

In summary, cross‐conjugated polymers remain an underexplored yet richly promising domain in the field of organic semiconductors. Their structural versatility and functional adaptability position them as strong candidates for future innovations in organic electronics.

## Conflicts of Interest

The authors declare no conflicts of interest.
